# Computational Identification of Sex-Biased Biomarker MicroRNAs and Genes Associated with Immune Infiltration in Breast Cancer

**DOI:** 10.3390/genes12040570

**Published:** 2021-04-14

**Authors:** Eric W. Li, Yongsheng Bai

**Affiliations:** 1 Lakeside School, 14050 1st Ave NE, Seattle, WA 98125, USA; ericl22@lakesideschool.org; 2 Department of Biology, Eastern Michigan University, 441 Mark Jefferson Hall, Ypsilanti, MI 48197, USA; 3 Next-Gen Intelligent Science Training, Ann Arbor, MI 48105, USA

**Keywords:** microRNA (miRNA), immune infiltration, breast cancer, sex-biased genes, sex-biased miRNAs

## Abstract

MicroRNAs (miRNAs) perform their functions through targeting messenger RNAs (mRNAs). X chromosome-located (X-linked) miRNAs have a broad role in cell lineage determination, immune regulation, and oncogenesis. The regulating roles of miRNAs in cancer and immunity are often altered when aberrant expression happens. Sex-biased genes could contribute to cancer sex bias in the context of their expression change due to targeting miRNAs. How biological roles and associations with immune cell abundance levels for sex-biased gene-miRNA pairs in gender-related cancer (e.g., breast cancer) change due to the alteration of their expression pattern to identify candidate therapeutic markers has not been investigated thoroughly. Upon analyzing anti-correlated genes and miRNAs within significant clusters of 12 The Cancer Genome Atlas (TCGA) cancer types and the list of sex-biased genes and miRNAs reported from previous studies, 125 sex-biased genes (11 male-biased and 114 female-biased) were identified in breast cancer (BC). Seventy-three sex-biased miRNAs (40 male-biased and 33 female-biased) were identified across 5 out of 12 cancers (head and neck squamous cell carcinoma (HNSC), kidney chromophobe (KICH), kidney renal clear cell carcinoma (KIRC), kidney renal papillary cell carcinoma (KIRP), and lung adenocarcinoma (LUAD)). Correlation between the BC sex-biased genes and tumor infiltrating immune cell types was further evaluated. We found eight genes having high correlation with immune infiltration. Fifteen candidate female-biased BC genes targeted by 3 X-linked miRNAs (*has-mir-18hashsa-mir-221*, and *hsa-mir-224*) were pinpointed in this study. Our computational result indicates that many identified female-biased genes which have positive associations with immune cell abundance levels could serve as alternative therapeutic markers. Our analysis suggests that female-biased expression of BC candidate genes is likely influenced by their targeting miRNA(s).

## 1. Introduction

Sex differences exist widely in a variety of human tissues besides the reproductive system. Sex is one of the most important factors affecting many biological processes and has a profound impact on the development, progression, and drug response of various diseases, including coronavirus disease 2019 [1,2] and cancer [3,4]. Although extensive progress has been made to reveal the sex-biased patterns of cancer incidence, mortality, and responses to treatments, including the latest immunotherapies [5,6], the molecular causes underlying such sex disparities remain largely unknown.

The messenger RNA is an outcome of gene transcription and is essential to performing biochemical functions in the cell. Molecular differences between male and female samples, including mRNA expression, have been reported from The Cancer Genome Atlas (TCGA) tumor datasets [7]. MicroRNAs (miRNAs) are a family of small, noncoding RNAs that modulate gene expression at the posttranscriptional level by two known mechanisms, the degradation of target mRNA and the suppression of protein expression [8]. There is accumulating evidence for differential miRNA expressions between women and men across a variety of tissues, and the sex-biased expression of miRNAs could have functional implications [9]. The regulation of mRNA by miRNA is complex. A single miRNA can target many mRNAs, while many miRNAs are able to cooperatively target a single mRNA, in both degradation and inhibition contexts [9]. Thus, mRNA-miRNA interaction pairs have been studied in cancer data sets. The anti-correlated mRNA-miRNA pairs in the same tissue between normal and tumor samples from TCGA together with identified sex-biased mRNA and miRNA could provide us with unprecedented opportunities to investigate the sex-biased mRNA-miRNA pairs in different cancer types and their functions related to sex disparity in cancer.

In the present day, breast cancer (BC) is one of the most common female tumors diagnosed. Breast cancer can be categorized as invasive or non-invasive carcinoma. Several common breast cancer subtypes in females are HR+/HER2−, HR−/HER2−, HR−/HER2+, and HR+/HER2+. The histologic subtypes in breast cancer are known as infiltrating ductal carcinomas, papillary carcinoma, and lobular carcinoma. Male breast cancer is rare, comprising of less than 1% of all breast cancer [10]. Lobular carcinoma is extremely rare in males, even though it is the second most common BC subtype in females [11]. How the molecular mechanisms contribute to differences in mortality between sexes for gender-related cancers (e.g., BC) is not well-known.

In this study, we utilized anti-correlated mRNA-miRNA pairs identified in a recent publication [12] and human sex-biased genes and miRNAs published to date [13,14] to unravel the targeting pattern of sex-biased genes and miRNAs and their potential roles in tumorigenesis and tumor invasion. The prioritized miRNAs and their targeting female-biased genes which have positive correlations with six different immune cell abundance levels could serve as alternative therapeutic cancer markers to design personalized immunotherapy.

## 2. Materials and Methods

### 2.1. Characterization of Sex-Biased Genes and miRNAs with Anti-Correlation Relationships Reflected in TCGA Cancer Types

#### 2.1.1. Obtaining the Anti-Correlated miRNAs and Gene Pairs from Previous Studies

We obtained the mRNA-miRNA pairs in the significant clusters (FDR < 0.1) identified for 15 TCGA cancer types from Dai et al. [12]. The 15 TCGA cancer types studied are bladder urothelial carcinoma (BLCA), breast invasive carcinoma (BRCA or BC), colon adenocarcinoma (COAD), esophageal carcinoma (ESCA), head and neck squamous cell carcinoma (HNSC), kidney chromophobe (KICH), kidney renal clear cell carcinoma (KIRC), kidney renal papillary cell carcinoma (KIRP), liver hepatocellular carcinoma (LIHC), lung adenocarcinoma (LUAD), lung squamous cell carcinoma (LUSC), prostate adenocarcinoma (PRAD), stomach adenocarcinoma (STAD), thyroid carcinoma (THCA), and uterine corpus endometrial carcinoma (UCEC). Significant clusters were generated by the modified Louvain algorithm as described in the paper [12]. Correlation coefficient values between each mRNA-miRNA pair and their expression information were used to implement the Louvain algorithm [15]. Selected mRNA-miRNA pairs in these clusters hold anti-correlation relationship between tumor and normal samples. The information regarding these pairs is reported in the study [12].

#### 2.1.2. Identification of Sex-Biased Genes and miRNAs Using Data from Existing Literature

We investigated sex-biased tendency for genes and miRNAs selected from the above step. Sex-biased genes and miRNAs are defined as having a significantly higher expression in one sex over the other sex. Existing studies [13,14] reported the sex-biased genes and miRNA results covering 12 TCGA cancer type datasets or corresponding to the tissue type for each said cancer: BLCA, BRCA (BC), HNSC, KICH, KIRC, KIRP, LIHC, LUAD, LUSC, PRAD, STAD, and THCA.

We wrote a suite of R programs (R3.6, downloaded on 20 July 2020; Available online: https://www.bioconductor.org/, accessed on 20 July 2020), which took the genes and miRNAs associated with TCGA cancers listed in the significant clusters of Dai et al. [12] and compared them to the sex-biased genes found by Guo et al. [14] and the sex-biased miRNAs from Cui et al. [13]. The common sex-biased genes and miRNAs were singled out.

#### 2.1.3. Analysis and Interpretation of the Sex-Bias Distribution Results

Custom R programs were written to examine the male-to-female ratio, density of fold change, and average fold change of the sex-biased genes and miRNAs. The proportions of male-biased to female-biased genes/miRNAs for each available cancer type were calculated. All figures were created with scripts programmed in R or Excel.

### 2.2. Prioritization of Female-Biased Breast Cancer Candidate Genes Correlated with Tumor-Infiltrating Immune Cell Types

#### 2.2.1. Analysis of the Association between Tumor-Infiltrating Immune Cells and Expression for Female-Biased Genes in BC

TIMER2.0 [16], a visualization tool of tumor-infiltrating immune cells for TCGA dataset, was employed to study the association between immune infiltration and gene expression for sex-biased genes in the context of six immune cell types: B cell, dendritic cell, macrophage, neutrophil, CD4+ T cell, and CD8+ T cell. The female-biased genes and their association with each immune cell type were evaluated.

#### 2.2.2. Categorization of Gene Ontology Terms and Pathways Enriched for Selected Female-Biased Genes with Positive Correlation between Their Expression and Immune Cell Abundance Level in BC

Functional annotation for genes showing positive correlation in CD8+ T cell and B cell immune cell types were initially analyzed. These genes are putatively involved in responding to tumor immune infiltration by CD8+ T cells and B cells. The DAVID functional annotation tool [17] was employed to identify enriched gene ontology (GO) terms and pathways for the selected 45 genes. Homo sapiens was used as the background species, and enriched biological processes from significant clusters (*p*-value < 0.05) were reported.

#### 2.2.3. Selection of Female-Biased Breast Cancer Candidate Genes with High Correlation Scores

Each female-biased BC gene was assigned a correlation score, which is defined by the number of immune cells that the specific gene is positively correlated with. As there are six immune cell types, the highest possible correlation score for a gene is 6, and the lowest possible score is 0.

The average correlation of genes with a high correlation score in each immune cell type was calculated. Since TIMER2.0 gave correlation results from many different immune infiltrate scoring methods, to reduce the bias for different immune infiltrate scoring methods a scoring formula was created to calculate the average correlation value of each gene in the context of each immune cell type (Equation (1)).

(1) $$\mathrm{Average}\;\mathrm{correlation}=\frac{\sum_{\;}^{\;}\;\mathrm{pos}\;+\sum_{\;}^{\;}\;\mathrm{neg}}{\#\;\mathrm{of}\;\mathrm{scoring}\;\mathrm{methods}}$$

Equation (1) Average correlation formula. pos= the correlation value of every scoring method that gave a positive correlation between that specific gene and that specific immune cell type. neg= the correlation value of every scoring method that gave a negative correlation between that specific gene and that specific immune cell type.

### 2.3. Pinpointing of miRNAs Targeting Female-Biased Breast Cancer Candidate Genes with High Correlation Scores

#### 2.3.1. Identification of X-linked miRNAs Targeting Female-Biased Breast Cancer Candidate Genes with High Correlation Scores

To make sure that we have included all possible X-linked miRNAs associated with breast cancer, we investigated existing literatures focusing on studies about X-linked miRNAs [7,18,19,20,21,22,23,24,25,26]. Using a customized R program, we identified female-biased breast cancer genes and their X-linked targeting miRNAs to search against the inversely correlated mRNA-miRNA pairs from Dai et al. (2018) [12].

#### 2.3.2. Examination of Sex-Bias Pattern for Neighboring Genes of Female-Biased Breast Cancer Genes Targeted by X-Linked miRNAs

Under the assumption that the expression of female-biased BC candidate genes is regulated by targeting miRNAs, their neighboring upstream and downstream genes should not consistently be female-biased. We retrieved nearby upstream and downstream genes for the 114 female-biased genes in BRCA and examined their sex-biased expression. Specifically, the closest function of BEDTools [27] was used. Three out of 114 genes, *C1orf106*, *GYLTL1B*, and *GRAMD2* were manually searched due to the naming annotation discrepancy between the UCSC genome browser and our genes. The sex bias of the neighboring genes was determined by using the sex-biased gene list generated by Guo et al. (2018) [14]. The neighboring genes not included in the list generated by Guo et al. [14] were labelled with no sex bias tendency.

## 3. Results

### 3.1. Identification of Sex-Biased Genes and miRNAs in Significant Clusters

Information on the significant clusters for 15 TCGA cancer types is shown in Table 1. Out of 15 input TCGA cancer types, BC and LUAD give the greatest number of significant clusters and the largest size (# of genes and miRNAs) of clusters when compared to others [12]. Three cancers, COAD, ESCA, and UCEC do not have significant clusters reported.

In total, 125 sex-biased genes, 11 male-biased and 114 female-biased, were found from the significant clusters in 1 out of 12 cancers, which is BC (Supplementary Table S1). Seventy-three sex-biased miRNAs, 40 male-biased and 33 female-biased, were found from the significant clusters across 5 out of 12 cancers (HNSC, KICH, KIRC, KIRP, and LUAD) (Supplementary Table S2). A summary of the sex-biased genes and miRNAs found is shown in Table 2.

### 3.2. Distribution of Sex-Biased Genes and miRNAs

#### 3.2.1. Male to Female Ratio of Sex-Biased miRNAs in Different Cancers

**Figure 1:**
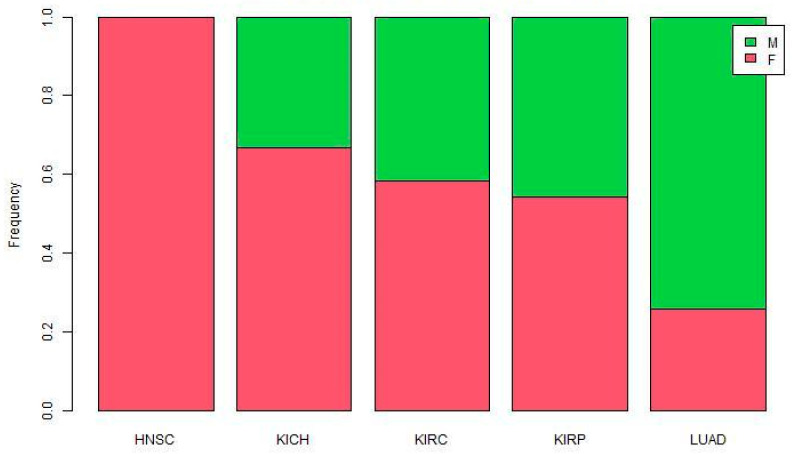
Proportion of sex-biased miRNAs in five TCGA cancers.

The ratio of male-biased to female-biased miRNA was not consistent across cancers (Figure 1). All reported miRNAs in HNSC are female-biased. The proportion of male-biased miRNA is high in LUAD, and low in other cancers.

#### 3.2.2. Fold Change of Sex-biased Genes and miRNAs

**Figure 2:**
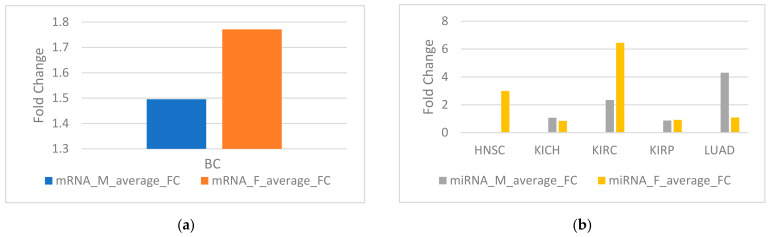
(**a**) Average fold change (FC) for sex-biased genes in breast cancer (BC). (**b**) Average FC for sex-biased miRNA in five TCGA cancers.

Fold change (FC) of a gene is defined as the average expression of the gene calculated in tumor samples divided by normal samples. The average fold change is defined as taking the average of the fold changes of all genes. The average fold change of female-biased genes in BC is higher than that of male-biased genes (Figure 2a). The average fold change of male-biased miRNA is higher than female-biased miRNA in LUAD, but the average fold change of female-biased miRNA is higher than male-biased miRNA in KIRC (Figure 2b).

#### 3.2.3. Density of Fold Change of Sex-Biased Genes and miRNAs

**Figure 3:**
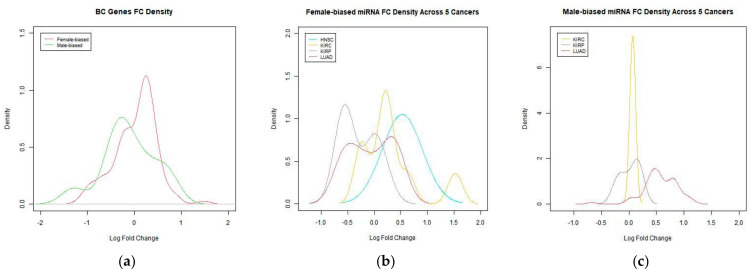
(**a**) FC density of genes in BC. (**b**) FC density of female-biased miRNA. (**c**) FC density of male-biased miRNA.

In BC, female-biased genes are upregulated, with the highest density around log FC 0.5. Male-biased genes are slightly down regulated (Figure 3a). Female-biased miRNAs in HNSC are upregulated, while female-biased miRNAs in KIRP are downregulated (Figure 3b). Male-biased miRNAs in LUAD are upregulated (Figure 3c). No male-biased miRNAs were found for HNSC and KICH.

### 3.3. Association between Immune Infiltration and Gene Expression for Female-Biased Genes in BC

TIMER2.0 [16] analysis results of the association between immune infiltration and gene expression for our selected female-biased genes focused on CD8+ T cell and B cell immune cell types initially. Both cell types give similar correlation trends. Indeed, the majority of female-biased genes (45 out of 58) show positive correlation between their expression and immune cell abundance level in both cell types, which could imply strong host immune reaction to female-biased cancer antigens. More than half of the genes (26 out of 45) showed positive correlation in both cell types for exhibiting higher expressions in tumor samples than in normal samples. Specifically, two breast cancer risk genes in the 26 identified genes, *RUNX3* and *CXCR6*, have high positive correlation in both immune cell types and higher expressions in tumor samples than normal samples, suggesting that these genes could act as antigens on tumor cells in breast cancer.

We also extended the analysis to check for correlation for expression of BC female-biased genes with immune cell abundance level in four additional immune cell types (dendritic cell, macrophage, neutrophil, and CD4+ T cell). The results are shown in Table 3 and Supplementary Table S3.

#### 3.3.1. Functional Annotation for Selected Female-biased Genes in BC

Functional annotation was done for the 45 genes (Supplementary Table S4) showing positive correlation to immune cell abundance level in both CD8+ T cell and B cell types. The major GO terms in significantly enriched functional annotation clusters are “membrane,” “cell membrane,” “receptor,” “domain: SH2,” and “transcription from RNA polymerase II promoter” (Table 4). The genes sharing these GO terms have been previously shown to be involved in breast cancer prognostic process (e.g., *CXCR6, KIT, RUNX3*) and also immune cell regulation (e.g., *CD8B*, *DAPP1, LY75*).

#### 3.3.2. Female-Biased Cancer Candidate Genes with High Correlation Scores

**Figure 4:**
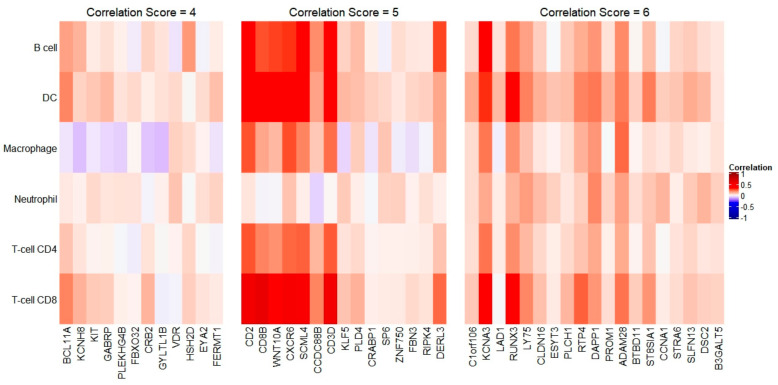
Association of immune cell infiltration and female-biased genes in BC. Only genes with a positive correlation score identified in four or more immune cell types were selected. Boxed regions signify candidate genes with positive correlation in most immune cell types.

The association between immune cell infiltration and female-biased genes in BC were also investigated. Out of 114 total female-biased genes, 19 had a correlation score of 6, 15 had a correlation score of 5, and 12 had a correlation score of 4. Average correlation of genes *CB3D*, *CD2*, *CD8B*, *CXCR6*, *KCNA3*, *RUNX3*, *SCML4*, and *WNT10A* is high across all immune cell types, suggesting that these genes could act as antigens on tumor cells in breast cancer (Figure 4).

### 3.4. Selection of Female-Biased mRNA-miRNA Pairs in BC

We acquired 29 X-linked miRNAs from published studies, as mentioned in the Materials and Methods section. Ten of these X-linked miRNAs were related to breast cancer [18,22,23,25,26] (Supplementary Table S5). Under the assumption that miRNAs differentially expressed in females of different ages are highly likely female-biased, we searched such a miRNA list against 96 differentially expressed miRNAs associated with breast cancer reported in the study [28]. We only found one miRNA (*hsa-mir-502*) meeting the criteria.

We obtained 114 female-biased genes in BC using our bioinformatic methods. Upon searching through the inversely correlated gene-miRNA pairs in BC from Dai et al. [12] for the pair relationship of 10 X-linked miRNAs related to breast cancer and the 114 female-biased genes in BC, we found 16 such gene-miRNA pairs (15 genes and 3 miRNAs), as shown in Table 5.

### 3.5. Differential Expression Tendency of Neighboring Genes of Female-Biased Genes inBC

Using BEDTools [27], neighboring upstream and downstream genes for the 114 previously obtained female-biased BC genes were examined and checked for their differential expression patterns.

The average expression of the neighboring genes was checked using gene expression data. The fold change of these genes, the average expression of the gene in tumor samples divided by normal samples, was studied. Out of all the closest upstream and downstream genes checked, eight upstream genes were in the same differential expression direction from the query gene. Ten downstream genes were also in the same differential expression direction compared to the query gene (Table 6). The fold change distribution of the 15 query genes and their neighboring genes show different correlation patterns (Query FC R^2^ = 0.04, Upstream FC R^2^ = 0.17, Downstream FC R^2^ = 7 × 10^−5^). This result indicates that the targeting miRNAs for the female-biased BC genes likely play a role in regulating female-biased expression for their targeted genes. To make sure the neighboring genes are not targeted by the same targeting miRNAs, the anti-correlated targeting gene-miRNA pairs were also checked, and no cases were found in which the same miRNA targeted the query gene and its neighboring genes. This result conveys the targeting exclusiveness of the sex-biased miRNAs into female-biased BC genes in the context of neighboring genes’ co-regulation.

## 4. Discussion

It is critical to understand the role of sex differences if we want to thoroughly understand the physiology of humans and genetic causes for gender-oriented cancers. Our knowledge of sex differences is especially important for understanding the sex disparity of certain diseases such as BC (more female patients than males) and LUAD (more male patients than female). This study investigated a list of female-biased genes in BC.

The association between immune infiltration and gene expression of these female-biased genes in BC was examined. Eight female biased genes in BC that have high correlation to immune cell abundance level across six immune cell types were identified. These genes could act as therapeutic biomarkers in BC.

As a significantly higher number of females contract BC than males, studying the female-biased genes associated with BC and their targeting miRNAs can offer insight into the mechanisms that cause BC. We envision that continued investigation of female-biased genes and their targeting miRNAs and narrowing down a candidate gene list can benefit the BC research community.

It is useful to point out that, in our study, we did not focus on genes and miRNAs according to BC subtype, age, or stage. Since the data we used were de-identified (TCGA Level 3), histologic subtype information was not available. It would be very interesting to conduct further studies on male patient data, given that there is under representation of lobular and HER2 positive cancer subtypes for male BC patients.

To our knowledge, this is the first study conducted to determine the interaction between female biased miRNA and genes through co-bias examination of targeting miRNAs and their neighboring genes. Three sex biased miRNAs were found to be inversely correlated with female biased genes. Studies on *hsa-mir-224* indicated its role in promoting tumorigenesis in triple negative breast cancers [29]. Prognostic value of *hsa-mir-221* has been demonstrated in a few cancer types, including breast cancer [30]. *hsa-mir-18b* expression has been shown to be associated with ER− breast cancer that displays a high degree of inflammation, suggesting its role in the systemic immunological response in ER- breast tumors [31].

It is important to note that the supplementary lists of Cui et al. [13] and Guo et al. [14] had limited numbers of sex-biased genes and miRNAs. Thus, when our programs searched against these lists, sex-biased genes were only found in 1 out of 12 cancers and sex-biased miRNAs were only found in 5 out of 12 cancers. It will be useful if a larger validation list of sex-biased genes/miRNAs becomes available for research in the future.

The association between female-biased genes in BC and tumor immune infiltration was only done for six immune cell types. We assumed that the more methods that reported positive correlation between a gene and a specific immune infiltrating cell type, the higher significance it posed in oncogenesis. It will be crucial to test if these associations hold in other immune cell types.

## 5. Conclusions

In conclusion, we identified a list of female biased genes in BC and found 8 of them, including *CD2*, *CD3D*, *CD8B*, *CXCR6*, *KCNA3*, *RUNX3*, *SCML4*, and *WNT10A* have high correlation to immune cell abundance level across six immune cell types. In particular, two genes, *RUNX3* and *CXCR6*, have high positive correlation across six immune cell types and higher expressions in tumor samples than normal samples. These genes could act as antigens on tumor cells and can potentially be the targets for developing personalized cancer treatment.

Our investigation on sex bias pattern of X-linked miRNAs and their relationship with these female biased genes in BC identified 16 inversely correlated sex biased gene-miRNA pairs (15 genes and 3 miRNAs) in BC. Our results suggest that the female-biased BC genes’ sex-bias tendency does not seem to be supported by the evidence of the common transcriptional mechanism with their neighbor genes; instead, the targeting miRNAs for the female-biased BC genes could likely play a role in regulating female-biased expression for their targeted genes. These findings shed light on the mechanism of sex bias determination in the gender-oriented cancers.

## Figures and Tables

**Table 1:** Information of significant clusters for 15 The Cancer Genome Atlas (TCGA) cancer types.

Cancer Types	# of Significant Clusters	# of Genes and miRNAs in the Clusters (Each Cluster Separated by Comma)
BLCA	2	136, 69
BC	8	593, 500, 775, 527, 362, 628, 511, 366
COAD	0	0
ESCA	0	0
HNSC	4	188, 109, 125, 139
KICH	1	38
KIRC	8	219, 294, 306, 286, 477, 432, 297, 275
KIRP	4	192, 215, 145, 144
LIHC	1	61
LUAD	9	348, 634, 666, 446, 379, 641, 460, 884, 330, 908
LUSC	2	27, 39
PRAD	3	173, 175, 213
STAD	8	500, 270, 210, 294, 265, 434, 228, 425
THCA	4	147, 72, 60, 43
UCEC	0	0

Note: Three cancers (colon adenocarcinoma (COAD), esophageal carcinoma (ESCA), and uterine corpus endometrial carcinoma (UCEC)) do not have significant clusters reported.

**Table 2:** Summary of sex-biased genes in BC and miRNA in five TCGA cancers (head and neck squamous cell carcinoma (HNSC), kidney chromophobe (KICH), kidney renal clear cell carcinoma (KIRC), kidney renal papillary cell carcinoma (KIRP), and lung adenocarcinoma (LUAD).

Sex Bias	Gene	miRNA
Male-biased	11 (8.8%)	40 (54.8%)
Female-biased	114 (91.2%)	33 (45.2%)

Note: Percentages inside the parentheses are the proportion of the corresponding bias category.

**Table 3:** Percentage of BC female-biased genes with positive correlation for six immune cell types based on results from TIMER2.0.

Immune Cell Type	Percentage of Genes with Positive Correlation
Dendritic cell	63.2% (72/114)
Neutrophil	59.6% (68/114)
CD8+ T cell	50.9% (58/114)
B cell	43.9% (50/114)
Macrophage	42.1% (48/114)
CD4+ T cell	41.2% (47/114)

**Table 4:** Gene ontology (GO) terms of genes with positive correlation with different immune cell types.

Positive Correlation with Immune Cell Type	Major GO Terms from DAVID
CD8+ T cell and B cell	“membrane”, “cell membrane”, “receptor,” “domain: SH2”, “transcription from RNA polymerase II promoter”

**Table 5:** Selected female-biased genes in BC and X-linked miRNA related to breast cancer with inverse correlation.

mRNA	miRNA	T_CC	T_P	T_FDR	N_CC	N_P	N_FDR
*GPRIN2*	*hsa-mir-224*	0.175638	1.10 × 10^−6^	1.48 × 10^−5^	−0.52346	1.97 × 10^−7^	1.57 × 10^−5^
*FBN3*	*hsa-mir-224*	0.135792	0.000173	0.001257	−0.28208	0.008121	0.065481
*GPRIN2*	*hsa-mir-18b*	0.090508	0.012555	0.045481	−0.3694	0.00043	0.007796
*DSC2*	*hsa-mir-224*	0.184346	3.09 × 10^−7^	4.74 × 10^−6^	−0.54743	4.09 × 10^−8^	4.15 × 10^−6^
*BCL11A*	*hsa-mir-224*	0.197716	3.88 × 10^−8^	7.31 × 10^−7^	−0.5425	5.71 × 10^−8^	5.51 × 10^−6^
*KCNA3*	*hsa-mir-224*	0.094124	0.009423	0.03605	−0.33546	0.001492	0.019649
*ST8SIA1*	*hsa-mir-224*	0.20703	8.38 × 10^−9^	1.82 × 10^−7^	−0.50737	5.29 × 10^−7^	3.60 × 10^−5^
*CXCR6*	*hsa-mir-224*	0.210274	4.83 × 10^−9^	1.10 × 10^−7^	−0.39647	0.000144	0.003366
*RUNX3*	*hsa-mir-18b*	0.103734	0.0042	0.018587	−0.26933	0.01165	0.083606
*CD3D*	*hsa-mir-224*	0.098973	0.00632	0.026008	−0.3057	0.003984	0.039943
*FERMT1*	*hsa-mir-224*	0.221817	6.32 × 10^−10^	1.73 × 10^−8^	−0.55639	2.20 × 10^−8^	2.46 × 10^−6^
*C4A*	*hsa-mir-221*	−0.12912	0.000359	0.002346	−0.367207	0.000468	0.00831
*GRAMD2*	*hsa-mir-224*	0.257596	5.52 × 10^−13^	2.67 × 10^−11^	−0.56696	1.03 × 10^−8^	1.29 × 10^−6^
*PLEKHG4B*	*hsa-mir-224*	0.188081	1.76 × 10^−7^	2.85 × 10^−6^	−0.55428	2.55 × 10^−8^	2.79 × 10^−6^
*SLFN13*	*hsa-mir-224*	0.118989	0.001014	0.005676	−0.55342	2.71 × 10^−8^	2.93 × 10^−6^
*CD8B*	*hsa-mir-224*	0.116876	0.001247	0.00676	−0.42097	4.90 × 10^−5^	0.001447

Note: T_CC and N_CC are the mRNA and miRNA correlation coefficient in tumor and normal samples, respectively. T_P and N_P are the *p*-values of the tumor and normal samples, respectively. T_FDR and N_FDR are the FDR values of the tumor and normal samples, respectively. These values were calculated by using the methods mentioned in Dai et al. (2018) [12].

**Table 6:** Average expression of closest upstream and downstream genes of selected female-biased genes in BC.

Query Gene	Query Gene FC	Upstream Gene	Upstream Gene FC	Downstream Gene	Downstream Gene FC
*RUNX3*	1.070115	*MIR4425*	0	***CLIC4***	0.6956
*KCNA3*	0.88596	***CD53***	1.394738	***KCNA2***	0.137172
*GPRIN2*	0.806098	*LOC102724488*	0	*NPY4R2*	0.29892
*CD3D*	2.242477	***CD3G***	1.400305	*CD3E*	1.823305
*ST8SIA1*	0.502259	*C2CD5*	0.9289736	***CMAS***	1.383262
*SLFN13*	0.686176	*SLFN12L*	0.659786	*SLFN12*	0.726248
*DSC2*	0.894301	***DSC1***	0.135309	***DSC3***	0.531148
*FBN3*	4.680184	*CERS4*	1.9812753	***CCL25***	8.487113
*BCL11A*	0.797152	*PAPOLG*	0.816493	*MIR4432HG*	0
*CD8B*	1.703002	*RGPD2*	0	*CD8A*	1.511057
*FERMT1*	0.713849	*CASC20*	0	***LRRN4***	3.825036
*CXCR6*	1.464406	***LZTFL1***	0.902672	*XCR1*	1.150559
*PLEKHG4B*	1.2639	*LRRC14B*	1.273693	*LRRC14B*	1.273693
*C4A*	2.739965	***STK19***	1.613933	*CYP21A1P*	0
*C4A*	2.739965	***STK19***	1.613933	***TNXB***	0.071174
*GRAMD2*	0.549913	*PKM*	1.9346012	*SENP8*	0.9139357

Note: Bold genes indicate that the ratio of the fold change between this gene and the query gene is greater than 1.5. Zeroes represent missing data.

## Data Availability

The data presented in this study are available in [12,13,14].

## Supplementary Materials

The following are available online at https://www.mdpi.com/article/10.3390/genes12040570/s1, Table S1: List of sex-biased genes selected from BC clusters, Table S2: List of sex-biased miRNA selected from TCGA clusters, Table S3: Association between female-biased genes in BC and 6 different immune cell types, Table S4: Full Gene Ontology Results for Select Genes, Table S5: X-linked miRNAs obtained from published studies.
